# miR-27a Targeting *PIK3R3* Regulates the Proliferation and Apoptosis of Sheep Hair Follicle Stem Cells

**DOI:** 10.3390/ani13010141

**Published:** 2022-12-29

**Authors:** Mengqi Yu, Lanlan Li, Meng Liu, Lei Wang, Xiaoxiao Gao, Lisheng Zhou, Nan Liu, Jianning He

**Affiliations:** 1 College of Animal Science and Technology, Qingdao Agricultural University, Qingdao 266109, China; 2 Animal Husbandry Development Center of Pingyuan County, Dezhou 253100, China; 3 Agriculture and Rural Affairs Bureau of Lanshan District, Linyi 276003, China

**Keywords:** miR-27a, Hair follicle stem cells, *PIK3R3*, AKT/MTOR pathway, cell proliferation and apoptosis

## Abstract

**Simple Summary:**

The quality of wool is directly related to the development of hair follicles. The effect of hair follicles on hair growth is related to the processes of proliferation, differentiation and apoptosis of hair follicle stem cells (HFSCs). Therefore, research on HFSCs in agricultural animals is rapidly gaining attention. The microRNA miR-27a has been shown to be involved in several diseases, such as lung cancer, ischemic stroke, neurofibromatosis and acute myocardial infarction, but the role of miR-27a in hair follicle development has not been studied. Here, we revealed that miR-27a is involved in the proliferation and apoptosis of HFSCs through its inhibition of the AKT/MTOR signaling pathway by targeting *PIK3R3*. We identified an miR-27a/*PIK3R3* regulatory network with potential implications for hair follicle development and wool quality.

**Abstract:**

Micro RNAs are regulatory factors in tissue development, organ formation, cell growth, apoptosis and other biological processes. In particular, several miRNAs are related to the development of hair follicles. Here, we investigated the effect of the targeting of *PIK3R3* by miR-27a on the AKT/MTOR pathway and on the proliferation and apoptosis of hair follicle stem cells (HFSCs) in sheep. Knockdown of the expression of *PIK3R3* was found to significantly inhibit the proliferation and promote the apoptosis of HFSCs. Similarly, a miR-27a mimic significantly inhibited the proliferation and promoted the apoptosis of HFSCs. The miR-27a mimic was also shown to significantly inhibit the expression of *PIK3R3*, AKT, and MTOR and the phosphorylation of AKT and MTOR, while a miR-27a inhibitor increased the expression of these genes. The presence of an miR-27a binding site in the 3′ UTR of *PIK3R3* was identified by a bioinformatics analysis, and the interaction was verified with a dual-luciferase reporter assay. The expression of *PIK3R3* mRNA and protein was negatively correlated with the presence of miR-27a, which suggests that this interaction may be involved in the biological impacts on proliferation and apoptosis. Thus, this study demonstrates that miR-27a plays a potential role in the proliferation and apoptosis of sheep hair follicle stem cells by targeting *PIK3R3*, which can be used to design new methods to improve sheep wool.

## 1. Introduction

Hair follicle tissue grows continuously throughout the life of the body [1]. Hair follicles have multiple functions, including roles in protection, immunity and sensation. During the development of hair follicles, the proliferation and differentiation of hair follicle stem cells (HFSCs) play central roles. HFSCs have a strong proliferation ability and can differentiate into hair follicles, epidermis cells, and sebaceous glands, and they have become a hot field of biological and medical research [2]. However, most of the research regarding HFSCs has focused on their properties in mice and humans, and less research has been performed on agricultural animals such as sheep and goats.

In a previous sequencing analysis, the gene encoding phosphoinositide 3-kinase regulatory subunit 3 (*PIK3R3*) was identified as a candidate gene related to the growth and development of hair follicles [3]. The gene may be related to AKT and the MTOR signaling pathway, which has been shown to affect the proliferation and apoptosis of HFSCs. The AKT/MTOR pathway has been shown to be closely linked to the hair growth process [4]. Transcription of *PIK3R3* attenuates skin inflammation in mice with psoriasis [5], and *PIK3R3* gene has been identified as a key gene regulating hair growth in mice [6].

The expression of some genes is impacted by microRNAs (miRNAs), which are short-chain non-coding RNAs [7]. They can inhibit the expression of target genes by binding to the 3’ UTR region of mRNA [8,9]. Inhibition by miRNAs plays important roles in tissue development, organ formation, cell growth and apoptosis [10,11,12]. Specifically, several miRNAs have been shown to be related to the growth and development of hair follicles. For example, the expression of miR-203a-3p promotes loureirin A-induced HFSC differentiation by targeting Smad1 [13]. Similarly, miR-31-5p promotes proliferation and inhibits apoptosis of goat HFSCs by targeting the RASA1/MAP3K1 pathway [14]. Another miRNA, mir-149-5p, promotes β-catenin-induced goat HFSC differentiation [15], and overexpression of miR-29a inhibits the proliferation of HFSC and further affects hair loss [16].

The gene encoding another miRNA, miR-27a, was located on chromosome 19 and was shown to play a vital role in tumor development [17]. This miRNA has multiple functions and can target multiple genes and exert corresponding cellular effects by inhibiting the expression of target proteins. One function of miR-27a-3p is to modulate ferroptosis via the targeting of *SLC7A11* in non-small-cell lung cancer cells [18]. This miRNA also appears to suppress cerebral ischemia-reperfusion injury by targeting *FOXO1* [19]. The upregulation of miR-27a-3p and miR-27b-3p was found to suppress the expression of *NF1* in dermal HSC and ST88-14 cells [20], and *EIF4A3*-induced circ-BNIP3 aggravated hypoxia-caused injury of H9c2 cells through targeting the miR-27a-3p/BNIP3 pathway [21]. The expression of miR-27a has also been shown to regulate the PI3K-Akt-MTOR axis in human chondrocytes, and it may participate in the pathogenesis of osteoarthritis [22]. This miRNA is thought to relieve osteonecrosis of the femoral head and absorbance repression in glucocorticoid-induced human bone marrow stem cells by targeting the PI3K/AKT/MTOR pathway [23]. *FBXW7* was shown to be regulated by miR-27a, and this regulation inhibited proliferation through the PI3K/AKT pathway and influenced invasion-mediated epithelial–mesenchymal transition in an oral squamous cancer cell line [24]. Differential expression of miR-27a has been identified in the skin of white and brown Cashmere goats, and *WNT3A* and *KITLG* were shown to be negatively regulated by miR-27a in these animals [25].

Through transcriptome sequencing of the skin tissue of Aohan fine wool sheep at 90 days, 120 days and the first birth, our laboratory previously identified the interaction between miR-27a and *PIK3R3* as a regulatory network related to hair follicles [3], but the function of miR-27a in HFSCs of sheep has not been studied in depth. In the present study, we investigated the possibility that miR-27a directly targets *PIK3R3* and determined the impact of this interaction on the MTOR signaling pathway and the regulation of the proliferation and apoptosis of HFSCs.

## 2. Materials and Methods

### 2.1. Culture of HFSCs

The Aohan fine-wool sheep HFSC line was isolated in this laboratory. Skin taken from the neck was sterilized with 75% alcohol and then rinsed with PBS buffer. Hair follicles were dissected from the tissue using a stereo microscope. Trypsin (0.25%) was added to the centrifuge tube, and digestion proceeded at 37 °C for 15 min. DMEM/F12 medium containing 10% FBS was added to terminate digestion. The sample was centrifuged at 1000× *g* for 5 min, and the supernatant was discarded. After the hair follicle tissue was crushed, F12 culture medium was added again, and the mixture was passed through a 200-mesh cytoscreener. The cells were again collected by centrifugation and then resuspended. Following inoculate into a culture bottle, the cells were cultured in DMEM medium containing 10% FBS and 1% penicillin/streptomycin at 37 °C, and the medium was replaced every 2 to 3 days [26].

### 2.2. Cell Transfection

Transfection was performed when the HFSCs had grown to 70% confluence. The plasmids pmirGLO-PIK3R3-WT and pmirGLO-PIK3R3-MUT and plasmids directing the expression of an miR-27a mimic, a negative control (NC) for the miR-27a mimic, an miR-27a inhibitor, an miR-27a inhibitor NC, an siRNA targeting *PIK3R3* (si-PIK3R3), and an NC for si-PIK3R3, were transfected with Lipofectamine^®^ 3000 Reagent (Invitrogen, Waltham, MA, USA) according to the manufacturer’s instructions [27].

### 2.3. Determination of Gene Expression

Primers used for real-time PCR quantification of mRNA expression (Table 1) were designed using an online tool from the NCBI and were synthesized by Tsingke Biotechnology Co., Ltd. (Beijing, China). RT-qPCR was performed in triplicate using the SYBR Green Pro Taq HS qPCR Kit (AG, Changsha, China). The reactions were performed in a real-time fluorescence-based quantitative PCR system (Bio-Rad, Hercules, CA, USA). Expression was normalized to the expression of *GAPDH*, and relative gene expression was calculated using the 2^−∆∆Ct^ method.

### 2.4. Dual Luciferase Reporter Assay

Identification of binding sites for miR-27a and *PIK3R3* was based on data sequenced previously [3] and was performed with the RNA22 miRNA target prediction tool http://cm.jefferson.edu/rna22 (accessed on 1 October 2021). The plasmids for the double luciferase experiment were synthesized by Tsingke Biotechnology Co., Ltd. (Beijing, China). The *PIK3R3* sequence was cloned into the PmirGLO vector (Promega, Madison, WI, USA) with both the wild-type (WT) 3′-UTR sequence and a sequence in which the 3′-UTR sequence was mutated (MUT). The 293T cells were seeded in 24-well plates, transfected with the noted plasmids, and incubated until they reached approximately 70% confluence. Cells were transfected with either pmirGLO-PIK3R3-WT or pmirGLO-PIK3R3-MUT and either the miR-27a mimic or the miR-27a mimic NC with Lipofectamine^®^ 3000 reagent according to the manufacturer’s instructions. After 48 h of incubation following transfection, the activity of luciferase was detected with a dual luciferase detection kit (Vazyme, Nanjing, China). Each experiment was performed in triplicate.

### 2.5. EdU and CCK-8 Assays

Cells were seeded at a density of 1 × 10^5^ in 24-well plates including a total of 6 groups with 3 replicates each. These 6 experimental groups were cells expressing si-PIK3R3, cells expressing a NC for the si-PIK3R3, cells expressing a miR-27a mimic, cells expressing a NC for the miR-27a mimic, cells expressing a miR-27a inhibitor and cells expressing a NC for the miR-27a inhibitor. After the cells were cultured overnight, they were labeled with EdU according to the instructions of the EdU cell proliferation detection kit (Beyotime, Shanghai, China). Then, the cells were incubated at 37 °C, 5% CO_2_ for 9 h before fixation and staining in the dark. The results were observed and photographed with a fluorescence microscope.

For CCK-8 assays, cells were seeded in 96-well plates at a density of 1 × 10^4^ with 100 μL of complete medium. The cells were transfected as described above, with each condition replicated 6 times. Following transfections, the cells were cultured at 37 °C, 5% CO_2_ for 48 h. Following this incubation, the cell culture medium was replaced, 10 μL of CCK-8 reagent was added to each well (Yeasen, Shanghai, China) and the cells were returned to the CO_2_ incubator. After 4 h, the absorbance value at a wavelength of 450 nm was measured using a microplate reader.

### 2.6. Detection of Apoptosis by Flow Cytometry

Cells (1 × 10^6^) were seeded in 6-well plates and transfected in triplicate as described above. After transfection, the cells were cultured for 48 h and then digested with trypsin without EDTA for 4 min. The cells were removed to a sterile tube and centrifuged at 300× *g* at 4 °C for 5 min. The pelleted cells were washed twice with pre-cooled PBS. The PBS was discarded, and the cells were resuspended in 100 μL of binding buffer. Then, annexin V-FITC (5 μL) and PI staining solution (10 μL) were added from commercially available kit (Yeasen, Shanghai, China), into the tube, which was shaken gently to mix. This mixture was incubated for 15 min in the dark at room temperature. Binding buffer (400 μL) was added, and the mixture was slowly pipetted to mix. The sample was placed on ice for immediate detection by flow cytometry. The experimental results were processed with FlowJo software.

### 2.7. Western Blot Assay

After transfected HFSCs were incubated for 48 h, the total cell protein was extracted and quantified with a BCA kit. The extracted protein samples were mixed with protein loading buffer (Beyotime, Shanghai, China) then heated in a boiling water bath for 5 min. The samples (10 μL per well) were loaded onto SDS-PAGE gels and subjected to electrophoresis followed by electrophoretic transfer to nitrocellulose membranes. For this transfer, the current was set to 250 mA, and the run time was typically 90 min, but the run time was 120 min for experiments involving detection of MTOR or p-MTOR. The membranes were blocked with 5% BSA for 1 h and incubated for 12 h with primary antibodies. The membranes were washed 3 times for 5 min with TBST buffer then were incubated with secondary antibody for 1 h and washed with TBST. Band densities were analyzed with Image J software. The rabbit primary antibodies used in this study were: GAPDH (1:1000, AC027, ABclonal), *PIK3R3* (1:500, A17112, ABclonal), AKT (1:500, bs-0115R, Bioss), p-AKT (1:500, bs-2720R, Bioss), MTOR (1:500, A2445, ABclonal), and p-MTOR (1:500, P0094, ABclonal). The secondary antibody was horseradish peroxidase-conjugated goat anti-rabbit IgG (1:2000, ab97051, ABcam).

### 2.8. Data Analysis

Data were analyzed with GraphPad v.8 (GraphPad Software Inc., San Diego, CA, USA). The data were expressed in the form of mean ± SD. Differences leading to *p* < 0.05 were considered to be statistically significant.

## 3. Results

### 3.1. PIK3R3 Regulates HFSC Proliferation and Apoptosis

**Figure 1:**
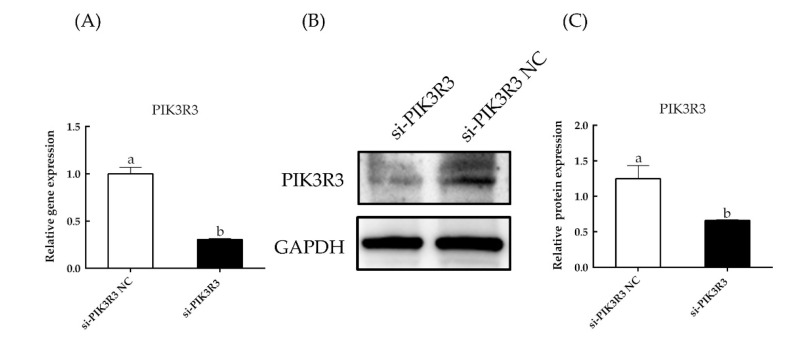
Effect of the siRNA-mediated knockdown of *PIK3R3* expression. HFSCs were transfected with si-PIK3R3 or a negative control (NC) construct. The different small letters (a, b) in the same graph indicate that the difference between groups is statistically significant (*p* < 0.05). (**A**) After 24 h of transfection, the expression of *PIK3R3* gene was detected by RT-qPCR. (**B**,**C**) Relative expression of proteins to GAPDH in HFSCs.

**Figure 2:**
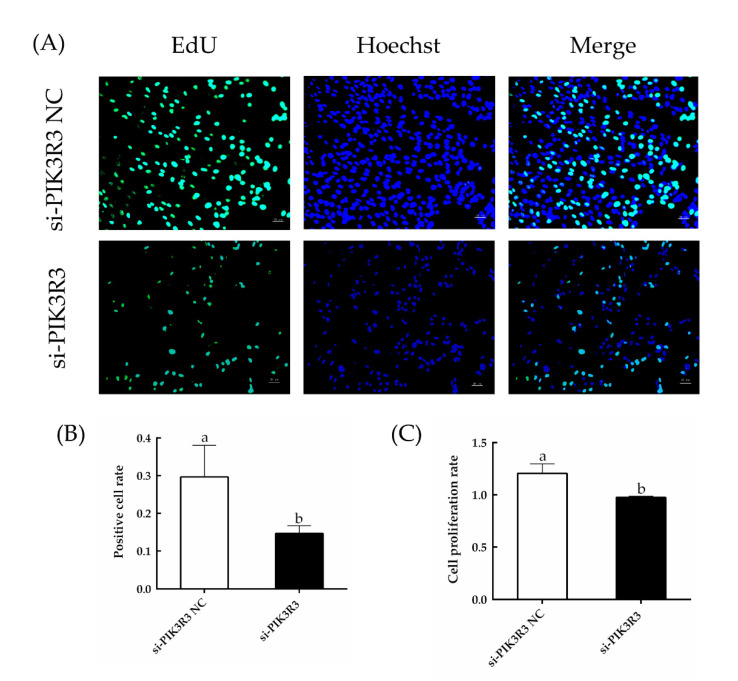
Effects of siRNA-mediated knockdown of *PIK3R3* expression on the proliferation of HFSCs. The different small letters (a, b) in the same graph indicate that the difference between groups is statistically significant (*p* < 0.05). (**A**) HFSC proliferation was measured by EdU assays 24 h after transfection of si-PIK3R3 or control. (**B**) The rate of EdU-positive staining was quantified and is expressed as the number of EdU-positive cells divided by the total number of cells. (**C**) The proliferation of HFSC cells transfected with si-PIK3R3 or control was measured with CCK-8 assays.

**Figure 3:**
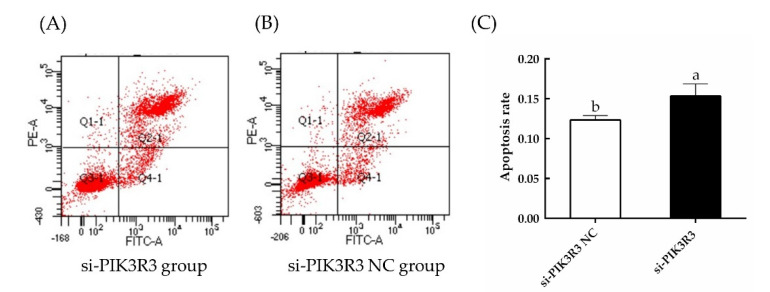
The effects of siRNA-mediated knockdown of *PIK3R3* expression on apoptosis were monitored by flow cytometry. The rate of apoptosis was measured by annexin V−FITC and PI staining of cells transfected with si-PIK3R3 (**A**) and an si-PIKR3 NC construct (**B**). (**C**) Quantification of the flow cytometry results from panels (**A**,**B**). The different small letters (a, b) in the same graph indicate that the difference between groups is statistically significant (*p* < 0.05).

As shown in Figure 1A and in Supplementary Figure S1, the expression levels of *PIK3R3* were significantly decreased in cells transfected with si-PIK3R3 as compared to the si-PIK3R3 NC group (*p* < 0.05). As compared with control, the cells transfected with si-PIK3R3 also had significantly reduced expression of *PIK3R3* protein (*p* < 0.05; Figure 1B,C). Similarly, EdU cell proliferation assays and CCK-8 cell viability assays (Figure 2) showed that the cell proliferation rate in cells transfected with si-PIK3R3 decreased significantly compared to control (*p* < 0.05). The apoptosis rate in si-PIK3R3-transfected cells increased significantly (*p* < 0.05; Figure 3), further suggesting that the *PIK3R3* gene positively regulates the proliferation of HFSCs.

**Figure 4:**
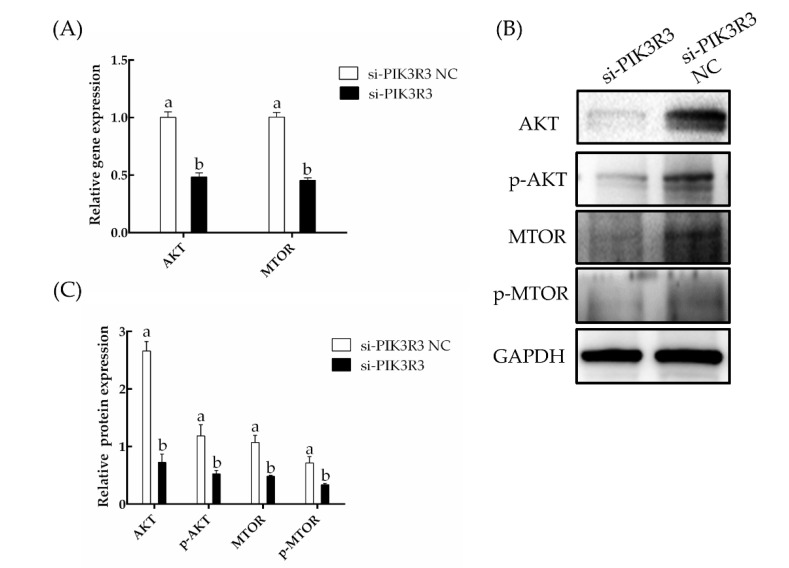
Effect of interfering with *PIK3R3* expression on AKT/MTOR signaling pathway. The different small letters (a, b) in the same graph indicate that the difference between groups is statistically significant (*p* < 0.05). (**A**) After 24 h of transfection, the expression of *AKT* and *MTOR* gene was detected by RT-qPCR. (**B**,**C**) Relative expression of proteins to GAPDH in HFSCs.

As can be seen in Figure 4A, transfection of si-PIK3R3 led to significantly lower levels of expression of *AKT* and *MTOR* mRNA compared to control, as determined by RT-qPCR (*p* < 0.05). Consistent with these results, cells treated with si-PIK3R3 also exhibited significantly lower levels of AKT protein, p-AKT, MTOR protein and p-MTOR (*p* < 0.05; Figure 4B,C, Supplementary Figure S1). These results suggest that *PIK3R3* supports proliferation and viability of these cells by enhancing the activity of a pathway involving AKT and MTOR that inhibits apoptosis.

### 3.2. The miRNA miR-27a Targets PIK3R3 and Downregulates the AKT/MTOR Pathway

**Figure 5:**
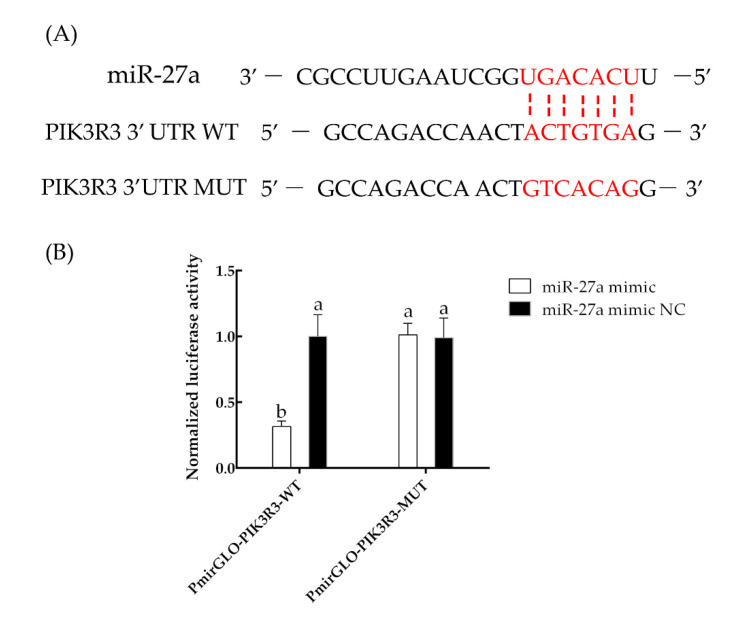
The miRNA miR-27a targets *PIK3R3* mRNA. (**A**) Sequence alignment of miR-27a and the 3′-untranslated region (UTR) of *PIK3R3* as determined with RNAv22. The different small letters (a, b) in the same graph indicate that the difference between groups is statistically significant (*p* < 0.05). (**B**) Luciferase activities in cells transfected with a luciferase plasmid containing the wild-type (WT) or mutated (MUT) PIK3R3 3′ UTR and either the miR-27a mimic or a negative control (NC).

A dual luciferase assay was employed to investigate the interaction of miR-27a with the *PIK3R3* gene. The miR-27a mimic was transfected with either a reporter containing the WT PIK3R3 3′ UTR or a reporter containing a mutated version of the 3′ UTR sequence (Figure 5A). The luciferase activity was significantly decreased in cells transfected with a miR-27a mimic and the luciferase reporter with the WT PIK3R3 3′ UTR compared to that of cells transfected with a NC miR-27a and the WT PIK3R3 3′ UTR plasmid (*p* < 0.05). The luciferase activities of cells transfected with the luciferase reporter with a mutated PIK3R3 3′ UTR and either the miR-27a mimic or the miR-27a NC were not significantly different (*p* < 0.05; Figure 5B).

### 3.3. Overexpression of miR-27a Inhibits HFSC Proliferation

**Figure 6:**
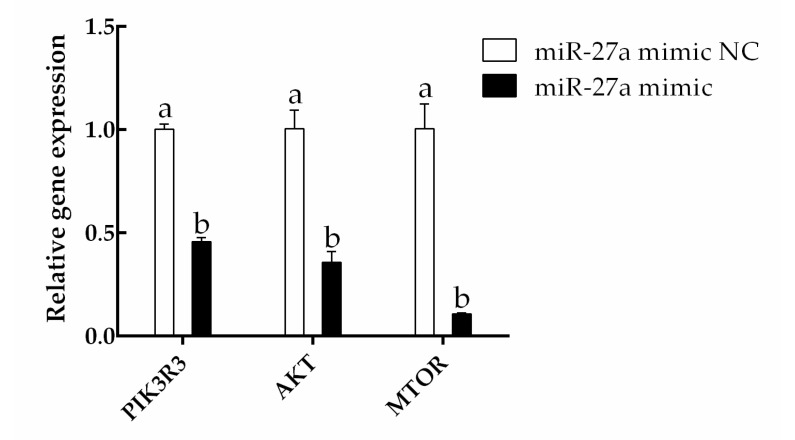
An miR-27a mimic decreases the expression of *PIK3R3*, *AKT*, and *MTOR*. HFSCs were transfected with the noted siRNAs, and gene expression was determined by RT-qPCR. The different small letters (a, b) in the same graph indicate that the difference between groups is statistically significant (*p* < 0.05).

The expression of *PIK3R3* was found to be significantly lower in cells transfected with an miR-27a mimic than in cells transfected with an miR-27a mimic NC (*p* < 0.05). In addition, the expression levels of *AKT* and *MTOR*, which are regulated by *PIK3R3*, were also found to be significantly reduced by the siRNA (*p* < 0.05; Figure 6). These results suggest that miR-27a negatively regulates the expression of *PIK3R3*.

**Figure 7:**
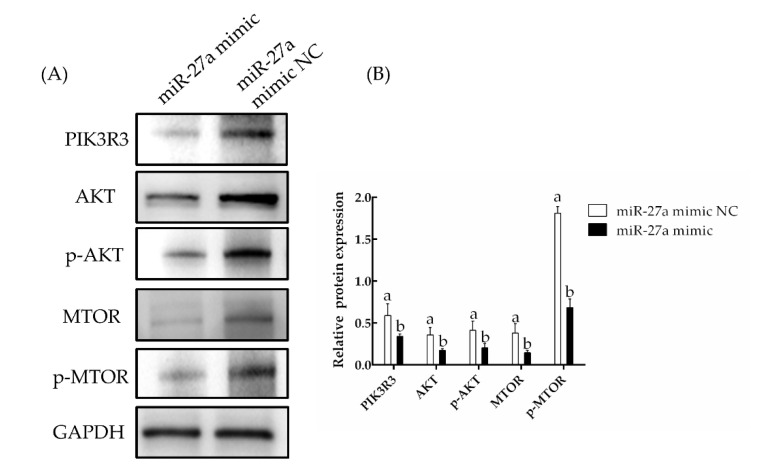
The effect of miR-27a mimic transfection on the expression and activation of proteins in the MTOR/AKT signaling pathway was determined by Western blotting. (**A**) Proteins were extracted 48 h after transfection with an miR-27a mimic or a negative control (NC) and analyzed with the noted antibodies. (**B**) The band densities in panel (**A**) were quantified with Image J. The different small letters (a, b) in the same graph indicate that the difference between groups is statistically significant (*p* < 0.05).

The effects of miR-27a on expression at the protein level were assessed by Western blotting. Here, the expression of *PIK3R3* protein was found to be significantly lower in cells transfected with the miR-27a mimic than in cells transfected with the miR-27a mimic NC. Similarly, the expression of AKT and MTOR proteins as well as the phosphorylation of AKT and MTOR were found to be significantly reduced by miR-27a (*p* < 0.05; Figure 7, Supplementary Figure S2). These results demonstrate decreased mRNA expression; together, these results indicated that miR-27a negatively regulates the expression of *PIK3R3*.

**Figure 8:**
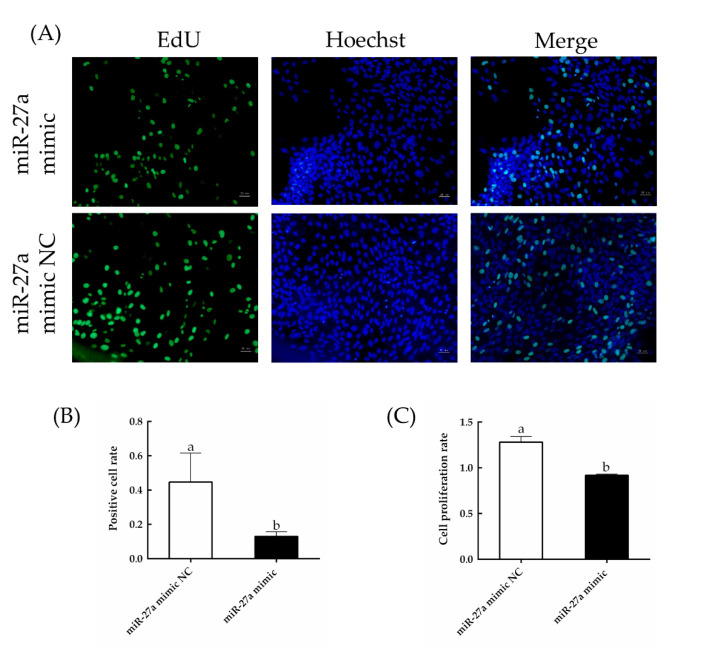
Effects of transfection of a miR-27a mimic on the proliferation of HFSCs. (**A**) HFSC proliferation was measured by EdU assays 24 h after transfection. (**B**) The rate of EdU-positive staining was quantified and is expressed as the number of EdU-positive cells divided by the total number of cells. (**C**) The proliferation of HFSC cells transfected with miR-27a or control was measured with CCK-8 assays. The different small letters (a, b) in the same graph indicate that the difference between groups is statistically significant (*p* < 0.05).

**Figure 9:**
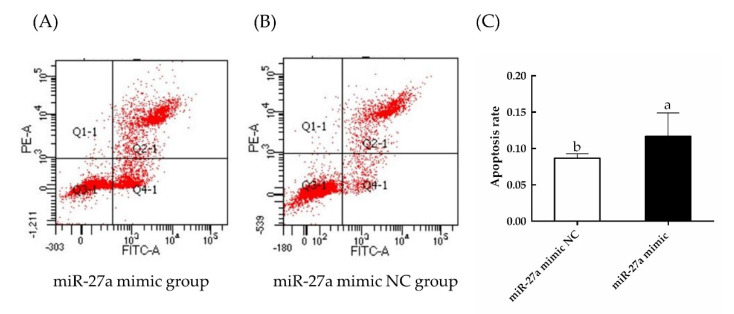
The effects of transfection of an miR-27a mimic on apoptosis were monitored by flow cytometry. The rate of apoptosis was measured by annexin V−FITC and PI staining of cells transfected with a miR-27a mimic (**A**) or a NC construct (**B**). (**C**) Quantification of the flow cytometry results from panels (**A**,**B**). The different small letters (a, b) in the same graph indicate that the difference between groups is statistically significant (*p* < 0.05).

As detected by EdU and CCK-8 assays (Figure 8), cell proliferation was significantly decreased by transfection of the miR-27a mimic compared to control (*p* < 0.05). In addition, as detected by flow cytometry (Figure 9), the rate of apoptosis was significantly increased (*p* < 0.05). These results are consistent with a model in which miR-27a reduces the expression of *PIK3R3*, thereby inhibiting the production and activation of AKT and MTOR, leading to an inhibition of proliferation of HFSCs and an increase of apoptosis of sheep HFSCs.

### 3.4. Interfering with miR-27a Promotes HFSC Proliferation

**Figure 10:**
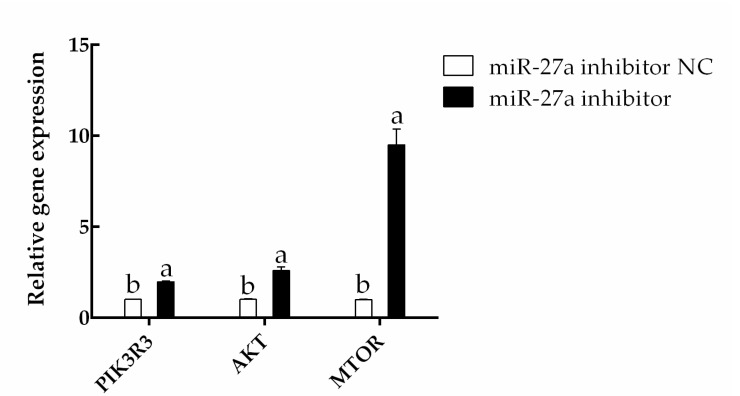
Transfection with an miR-27a inhibitor increases the expression of *PIK3R3*, *AKT*, and *MTOR*. HFSCs were transfected with the noted siRNAs, and gene expression was determined by RT-qPCR. The different small letters (a, b) in the same graph indicate that the difference between groups is statistically significant (*p* < 0.05).

**Figure 11:**
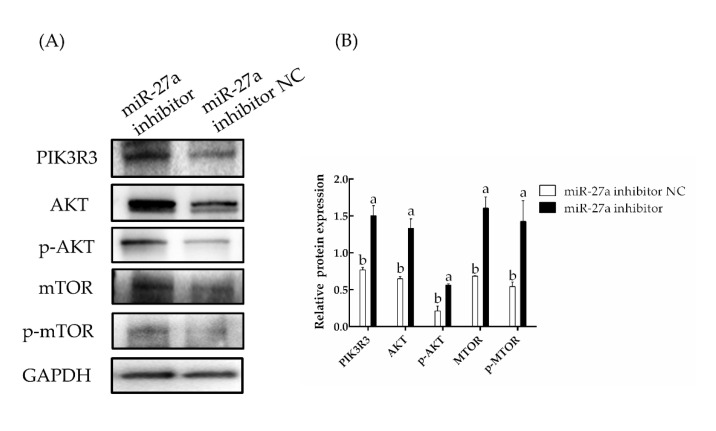
The effect of miR-27a inhibition on the expression and activation of proteins in the MTOR/AKT signaling pathway was determined by Western blotting. (**A**) Proteins were extracted 48 h after transfection with an miR-27a inhibitor or a negative control (NC) and analyzed with the noted antibodies. (**B**). The band densities in panel (**A**) were quantified with ImageJ. The different small letters (a, b) in the same graph indicate that the difference between groups is statistically significant (*p* < 0.05).

The effects of miR-27a were further investigated by transfecting an miR-27a inhibitor or a related NC. Here, the expression of *PIK3R3* was found to be significantly increased by transfection of the miR-27a inhibitor compared to control (*p* < 0.05). The expression of both *AKT* and *MTOR* were also raised significantly (*p* < 0.05), indicating that miR-27a has a negative regulatory effect on *PIK3R3* (Figure 10). The expression levels of related proteins were detected by Western blotting. Consistent with the qPCR results, the Western blotting experiments showed that the expression of *PIK3R3* protein was significantly increased by transfection of the miR-27a inhibitor compared to control (*p* < 0.05). Furthermore, the levels of AKT and MTOR protein and the phosphorylation of these proteins were also found to be increased by the inhibitor (*p* < 0.05; Figure 11, Supplementary Figure S3). These results again support the idea that miR-27a negatively regulates the expression of *PIK3R3*.

**Figure 12:**
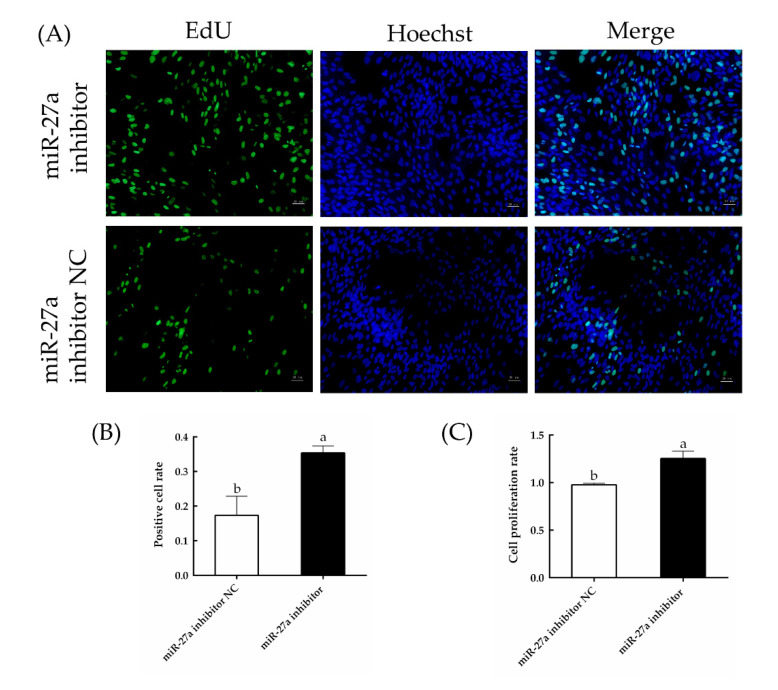
Effects of miR-27a inhibition on the proliferation of HFSCs. (**A**) HFSC proliferation was measured by EdU assays 24 h after transfection. (**B**) The rate of EdU-positive staining was quantified and is expressed as the number of EdU-positive cells divided by the total number of cells. (**C**) The proliferation of HFSC cells transfected with an miR-27a inhibitor or control was measured with CCK-8 assays. The different small letters (a, b) in the same graph indicate that the difference between groups is statistically significant (*p* < 0.05).

**Figure 13:**
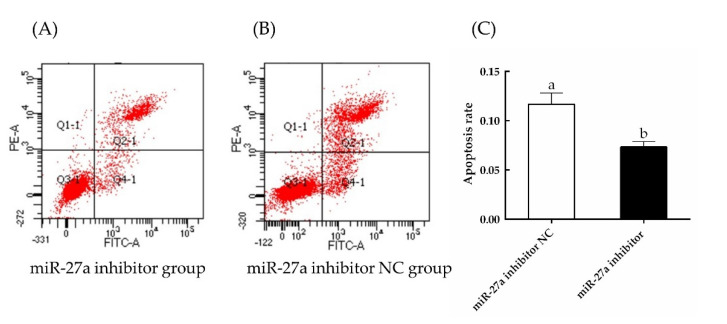
The effects of transfection of an miR-27a inhibitor on apoptosis were monitored by flow cytometry. The rate of apoptosis was measured by annexin V−FITC and PI staining of cells transfected with an miR-27a inhibitor (**A**) or an NC construct (**B**). (**C**) Quantification of the flow cytometry results from panels (**A**,**B**). The different small letters (a, b) in the same graph indicate that the difference between groups is statistically significant (*p* < 0.05).

EdU and CCK-8 assays (Figure 12) showed that the proliferation of HFSCs was significantly enhanced by the miR-27a inhibitor (*p* < 0.05), and the rate of apoptosis was clearly lower in cells transfected with the miR-27a inhibitor compared to control (Figure 13). Thus, interfering with miR-27a was shown to increase the expression of *PIK3R3*, which in turn increased the expression and activation of AKT and MTOR. Activation of this signaling axis increased the proliferation and reduced the apoptosis of sheep HFSCs.

## 4. Discussion

Aohan wool is produced by the development of hair follicles in a process that is vital to the wool spinning industry [28]. The development of hair follicles, which is known to be directly related to the quality of wool, is inseparable from the processes of proliferation, differentiation and apoptosis of HFSCs [29]. Consequently, the study of HFSCs has become an important aspect of biological research.

*PIK3R3* encodes a regulatory subunit of PI3K, which increases protein activity by binding to an activated protein tyrosine kinase, thereby affecting various cellular functions [30]. The expression of the *PIK3R3* gene has been found to be associated with skin lesions in human patients with hidradenitis [31], suggesting its role in skin development and hair growth. Similarly, as previously mentioned, *PIK3R3* is associated with skin development and hair growth in mice [6]. Despite these strong connections of *PIK3R3* to skin functions in humans and lab animals, there is no relevant research on sheep hair follicles. In the present study, roles of *PIK3R3* in proliferation and apoptosis were studied in HFSCs, and it was found that the proliferation of HFSCs was significantly inhibited, and the apoptosis was significantly enhanced by *PIK3R3* knock-down.

The PI3K/AKT/MTOR pathway is associated with skin cancer [32], perhaps through the involvement of the AKT/MTOR pathway on inhibiting the death of skin cells [33]. Activated *PIK3R3* can phosphorylate *AKT* and activate a signaling pathway involving *AKT* and *MTOR*, thus promoting cell growth, proliferation, protein synthesis and other processes [34]. In this study, interference with the expression of *PIK3R3* led to significant inhibition of the levels of expression of *AKT* and *MTOR*. In addition, this interference led to a significant reduction of the proliferation rate of HFSCs and to a significant increase of the rate of apoptosis. This suggests that *PIK3R3* regulates the proliferation and apoptosis of sheep HFSCs through the AKT/MTOR pathway.

The microRNA miR-27a has been shown to be important in multiple systems, including skin. Overexpression of miR-27a-5p has been shown to promote apoptosis of skin fibroblasts [35], and this miRNA can be used as a detection marker for screening and diagnosing skin squamous cell carcinoma [36], and it is associated with skin lesions [37]. It also participates in the expression of human keratinocytes [38]. Thus, this miRNA likely has similar functions in multiple cell types, as well as in multiple tissues and organs. Based on this, we speculated that miR-27a has a similar effect on sheep skin. Through experiments, we found that miR-27a significantly inhibited the proliferation of sheep hair follicle stem cells. A related miRNA, miR-27a-3p, has been found to affect the proliferation of human skin cells and hair follicle regeneration [39]. The result of this experiment is consistent with previous research results. Accordingly, through pre-sequencing results and bioinformatics predictions, we found that miR-27a may affect hair follicle development by targeting *PIK3R3*. A dual luciferase reporter assay using the *PIK3R3* 3′ UTR showed that addition of a miR-27a mimic into cells lowered the activity of this luciferase significantly compared to control; this indicated that miR-27a has a targeting relationship with *PIK3R3*. In addition, RT-qPCR and Western blot experiments showed that the expression of *PIK3R3* mRNA and protein was decreased by the miR-27a mimic, again, indicating that miR-27a may regulate hair follicle development by targeting *PIK3R3*. In the present study, it was found that the expression of miR-27a was negatively correlated with the expression of *PIK3R3* and the expression of *PIK3R3* was positively correlated with AKT and MTOR. This is consistent with previous studies that found *PIK3R3* activates the AKT/MTOR pathway to promote cell proliferation [34]. The experiments were carried out by EdU, CCK-8 and flow cytometry, and these results indicated that miR-27a negatively regulates the proliferation of HFSCs and positively regulates the apoptosis of HFSCs. Information from previous studies and the results described in this paper can provide a reference for the development of sheep breeds with better wool quality through analyses at the molecular level.

## 5. Conclusions

Our study indicated miR-27a targeted *PIK3R3*, and regulated sheep HFSC proliferation and apoptosis by the AKT/MTOR signaling pathway. This study provides the information to spur further research on the development of hair follicles and to accelerate the molecular breeding process in sheep.

## Figures and Tables

**Table 1:** The sequence of primers used in the study for mRNA quantitative real-time PCR.

Primer Name	Primer Sequences (5′-3′)	Gene ID	Product Size (bp)
*PIK3R3*	F: AGACTGGAGGGAGGTGATG	101123247	100
	R: AGTCATTGGCTTAGGTGGC		
*AKT*	F: GTCGCCCCTCAACAACTTCT	100294652	233
	R: CATCGTCTCCTCCTCCTCCTGCC		
*MTOR*	F: AGCATCTCTCCCCAAAGAACCTCA	100271659	221
	R: GGCCCTGGTCTCTTCATTCC		
*GAPDH*	F: AAGTTCAACGGCACAGTCA	443005	151
	R: ACCACATACTCAGCACCAGC		

## Data Availability

The datasets used and/or analyzed during the current study are available from the corresponding author on reasonable request.

## Supplementary Materials

The following supporting information can be downloaded at: https://www.mdpi.com/article/10.3390/ani13010141/s1, Figure S1: Changes of expression of *PIK3R3*, AKT, and MTOR proteins and struct in HFSCs, with GAPDH serving as a loading control. Figure S2: Changes of expression of *PIK3R3*, AKT, MTOR proteins and the level of p-AKT and p-MTOR upon transfection of a miR-27a mimic or a negative control (NC) in HFSCs, with GAPDH serving as a loading control. Figure S3: Changes of expression of *PIK3R3*, AKT and MTOR and the levels of p-AKT and p-MTOR proteins upon expression of a miR-27a inhibitor or a negative control (NC) in HFSCs, with GAPDH serving as a loading control.
