# Supplementary material for: miR-27a Targeting PIK3R3 Regulates the Proliferation and Apoptosis of Sheep Hair Follicle Stem Cells
Source: Animals (Basel). 2022 Dec 29;13(1):141. doi: 10.3390/ani13010141 (PMC9817964; doi:10.3390/ani13010141)
Supplement: Supplementary file 1 [file animals-13-00141-s001.zip › animals-2056437-supplementary.pdf]

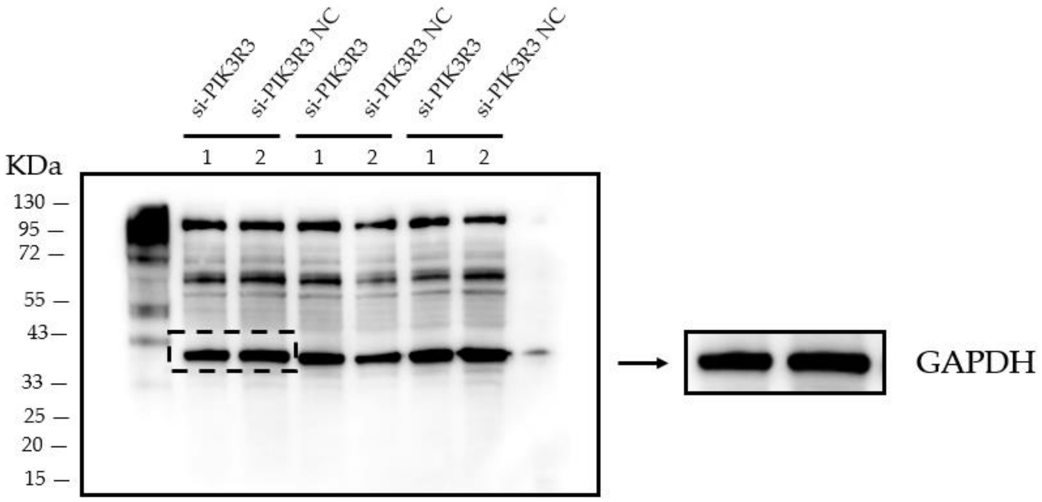
si-PIK3R3
si-PIK3R3 NC
si-PIK3R3
si-PIK3R3 NC
si-PIK3R3
si-PIK3R3 NC
KDa
1 2 1 2 1 2
130
95
72
55
43
33
25
20
15
GAPDH

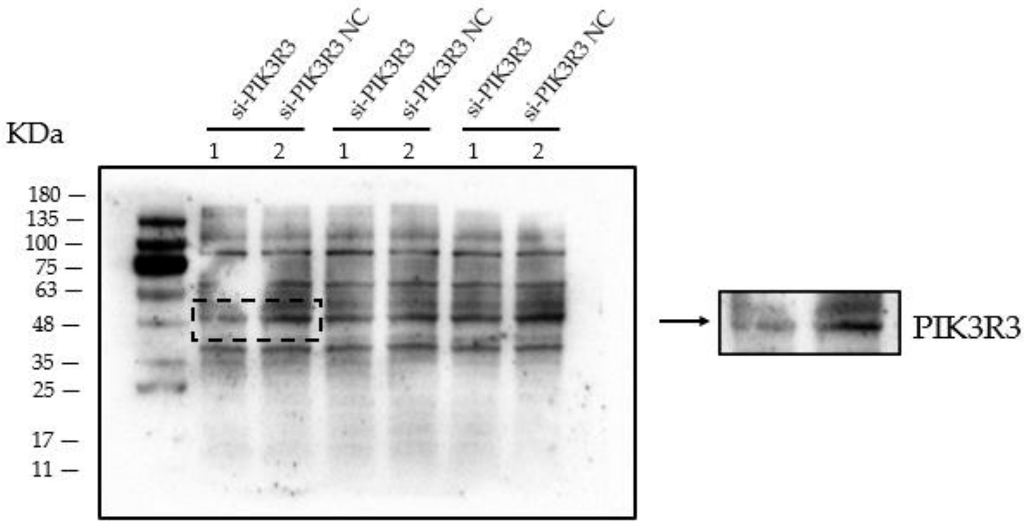
si-PIK3R3
si-PIK3R3 NC
si-PIK3R3
si-PIK3R3 NC
si-PIK3R3
si-PIK3R3 NC
KDa
1 2 1 2 1 2
180
135
100
75
63
48
35
25
17
11
PIK3R3

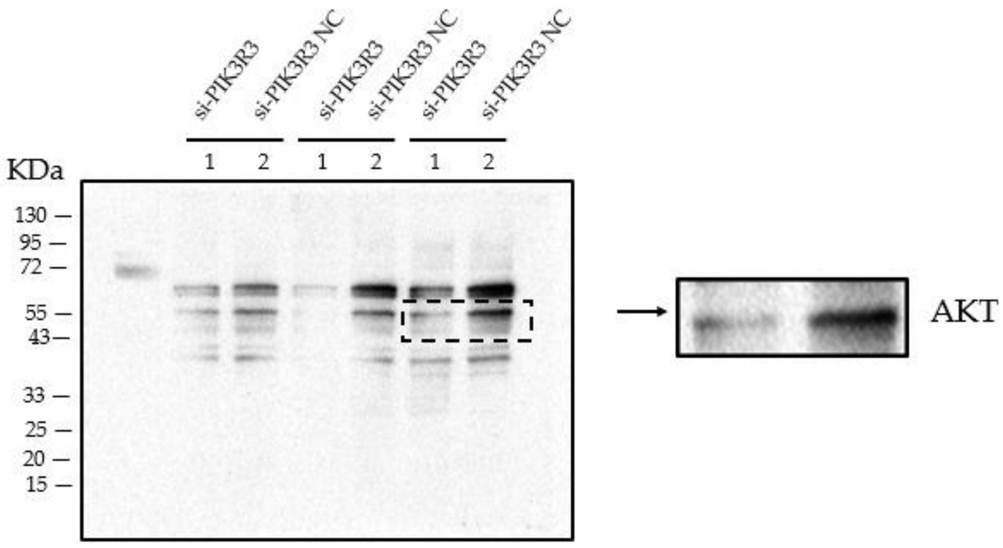
si-PIK3R3
si-PIK3R3 NC
si-PIK3R3
si-PIK3R3 NC
si-PIK3R3
si-PIK3R3 NC
KDa
1 2 1 2 1 2
130
95
72
55
43
33
25
20
15
AKT

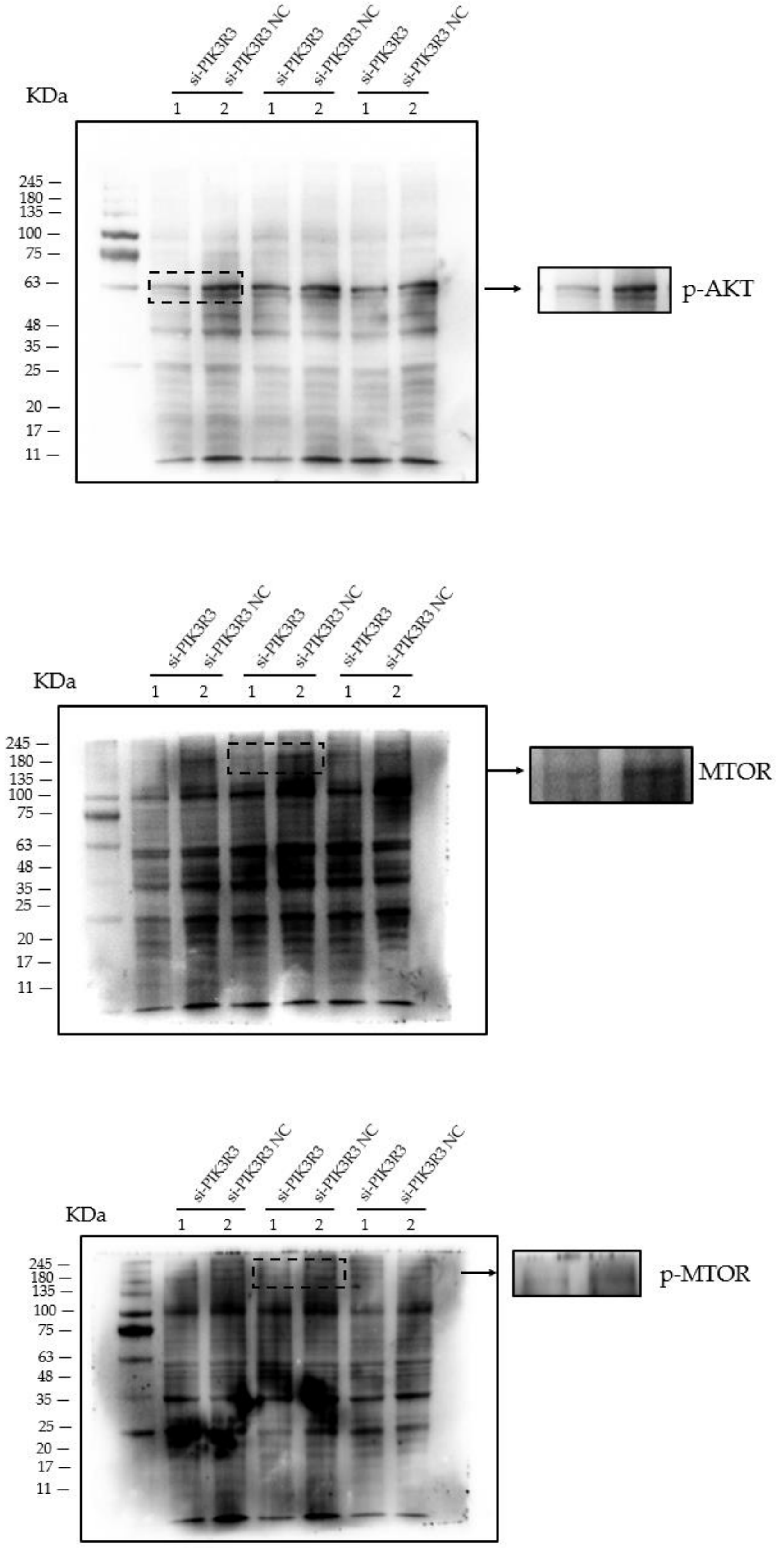

Figure S1. Expression changes of GAPDH, PIK3R3, AKT, p-AKT, MTOR and p-MTOR

proteins with si-PIK3R3 or a negative control (NC) construct in HFSCs.

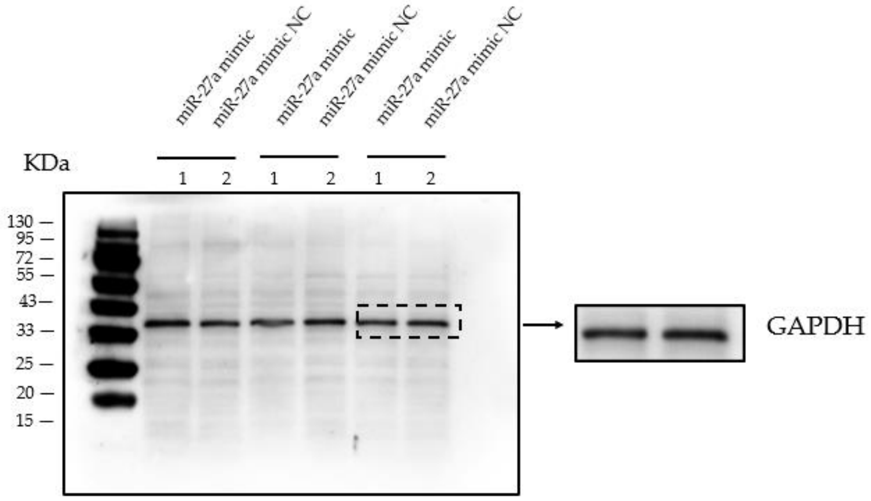

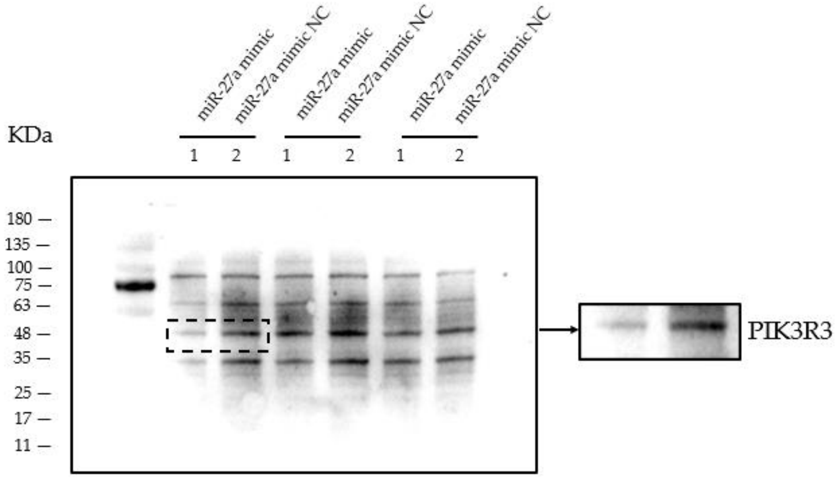

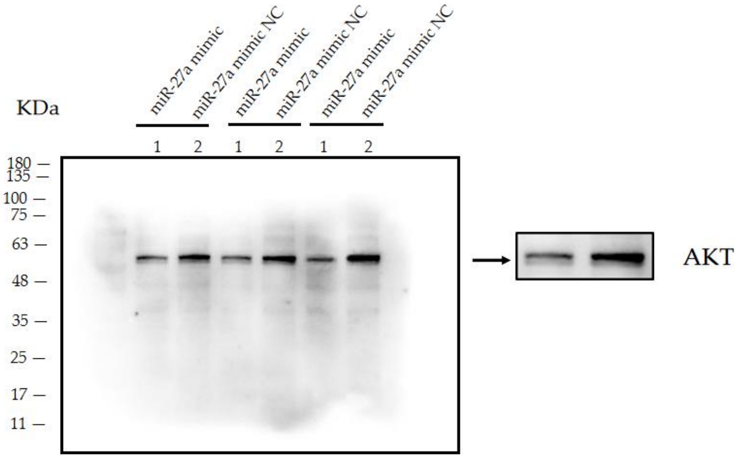

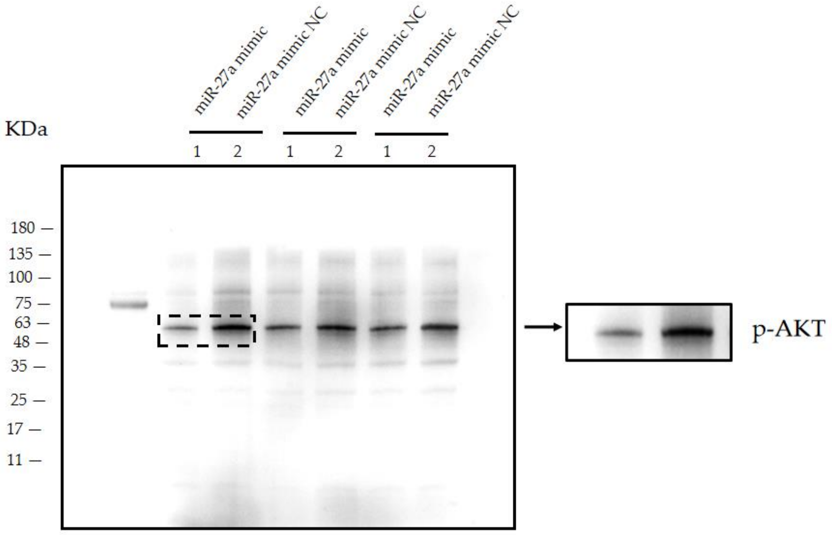

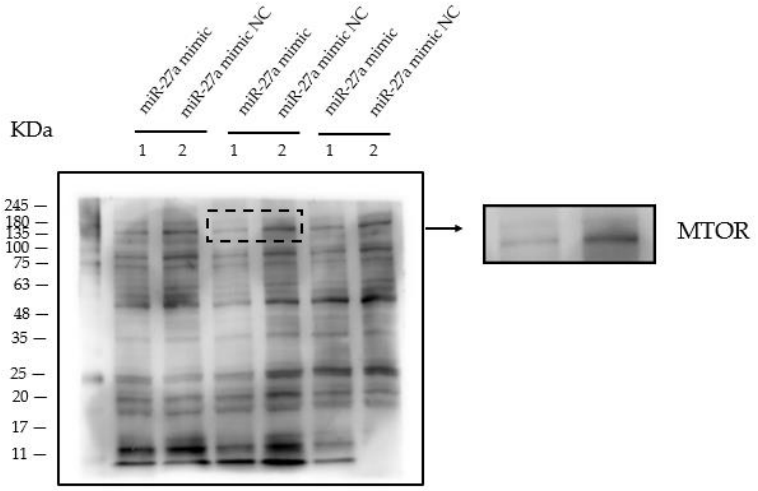

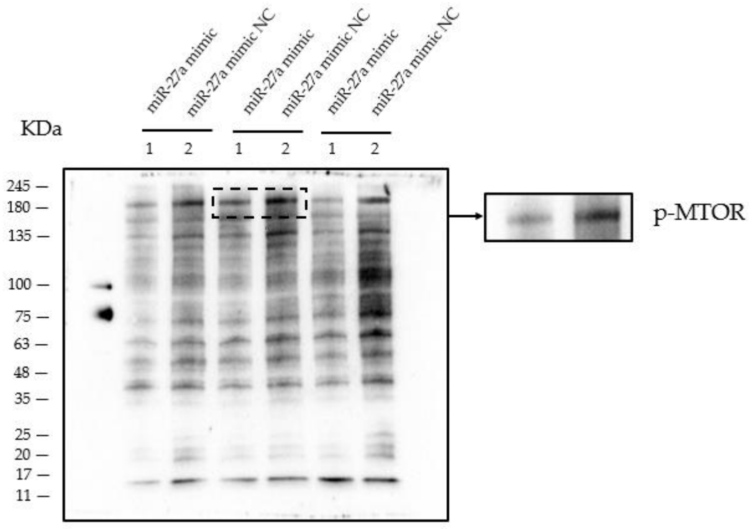

Figure S2. Expression changes of GAPDH, PIK3R3, AKT, p-AKT, MTOR and p-MTOR

proteins with a miR-27a mimic or a negative control (NC) in HFSCs.

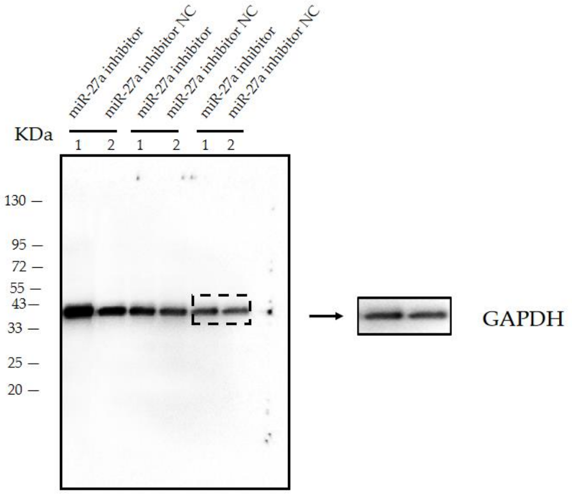

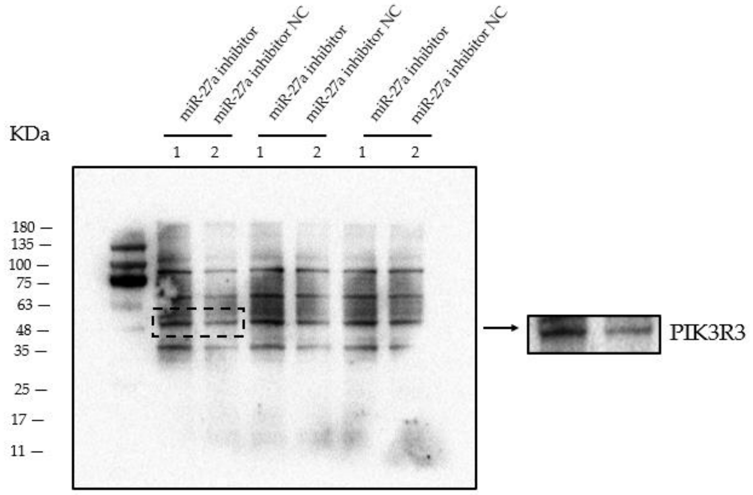

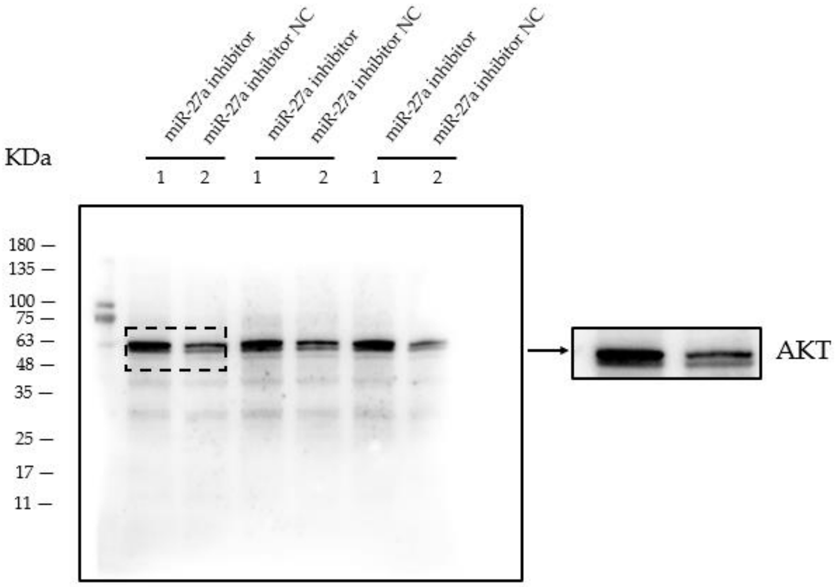
miR-27a inhibitor
miR-27a inhibitor NC
miR-27a inhibitor
miR-27a inhibitor NC
miR-27a inhibitor
miR-27a inhibitor NC
KDa
1
2
1
2
1
2
180
135
100
75
63
48
35
25
17
11
AKT

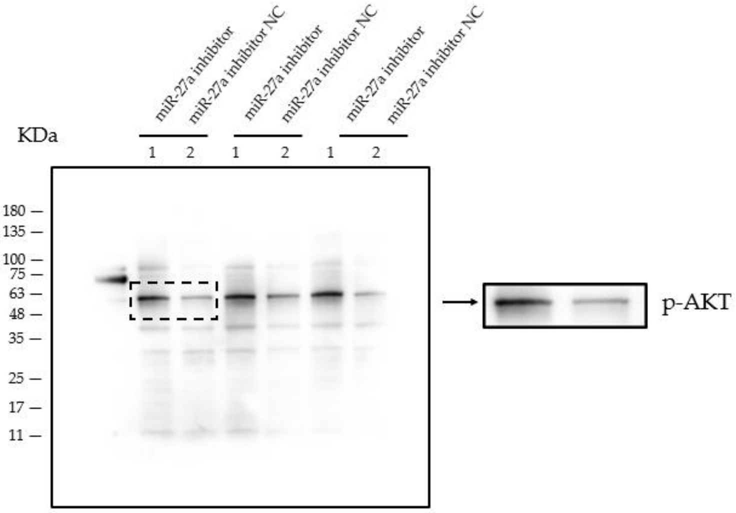
miR-27a inhibitor
miR-27a inhibitor NC
miR-27a inhibitor
miR-27a inhibitor NC
miR-27a inhibitor
miR-27a inhibitor NC
KDa
1
2
1
2
1
2
180
135
100
75
63
48
35
25
17
11
p-AKT

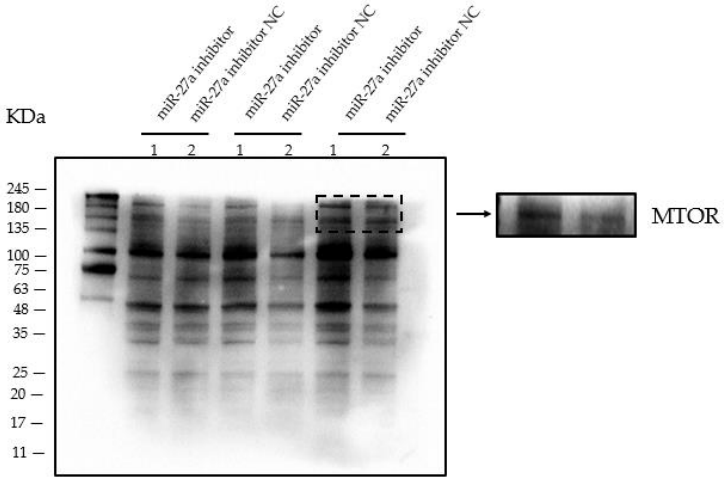

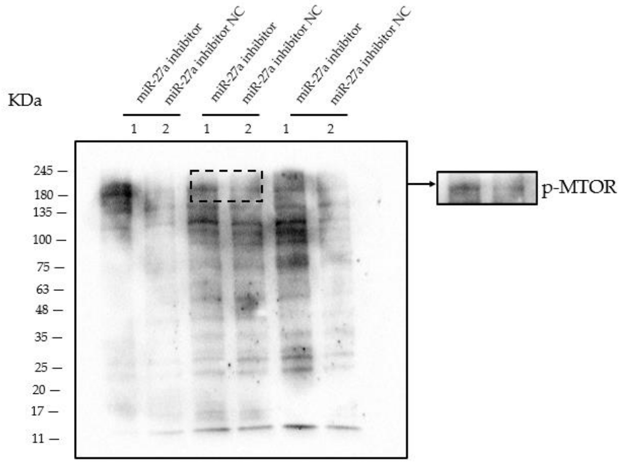

Figure S3. Expression changes of GAPDH, PIK3R3, AKT, p-AKT, MTOR and p-MTOR proteins with a miR-27a inhibitor or a negative control (NC) in HFSCs.
